# Operation for Carotid Aneurism

**Published:** 1830-04-01

**Authors:** 


### XXIX.

### SEQUEL OF "A CASE OF CAROTID ANEURISM, WHERE THE ARTERY WAS TAKEN UP ABOVE THE TUMOUR.*"

*Mauritius, Oct. 24th,* 1829.

SIR,

I had the honor, on the 9th of last June, to transmit you, by my friend Mr. Thomas Logan, the history of a case of carotid aneurism, which was operated on by me, ultra tumorem, on the 9th March, accompanied with a request, that you would be pleased to communicate it to the profession through whatever channel you might deem most eligible. I also promised that the result of the case should be communicated to you, a promise which I now fulfil.

Toward the conclusion of the case, it was stated "that the patient commenced to walk out of doors; that there was no discharge from the wound, which was on the eve of healing; that the tumour had entirely disappeared from the neck; and that, whatever fate was in reserve for the patient, the aneurism at least seemed to be cured."

June 10th. The patient complains of stiffness of throat, with difficult deglutition, which was attributed to his exposure in the open air after previous long confinement. An anodyne was exhibited and volatile liniment was used without good effect; on the contrary, the pain and stiffness extended to the right angle of lower jaw. A blister was applied and he was purged, with temporary relief.

13th. Slight purulent discharge from the wound; jaw continues stiff, with swelling of the parotid gland and discharge of saliva from the mouth. Emollient cataplasms, astringent gargles, &c.

18th. Wound entirely healed; other symptoms continue. Continue poultice, gargle, &c.

26th. Considerable purulent discharge from the mouth, proceeding from the parotid gland, in consequence of which the gland has become soft and decreased in size. From this period, tonic medicines, anodynes, and occasional aperients, (as symptoms indicated,) with a light nutritive diet, were given, and the cataplasm or hot fomentations and gargle continued. His health seemed to amend until two o'clock on the morning of the 3d July, when he was suddenly attacked with cough, and expectoration of florid blood to the extent of six ounces, accompanied with sinking of the pulse and every appearance of approaching dissolution. Sulphuric acid, mucilaginous mixture for the cough, &c. were given with some relief. The cough, however, continued, with expectoration at times mixed with blood, as also the discharge of purulent matter from the mouth. The debility increased until five o'clock of the evening of the 12th July, when he expired. At two the following day the body was inspected in the presence of a majority of the gentlemen who witnessed the operation, and several others.

*Dissection.* In order to expose the seat of the disease with the greatest accuracy; an incision was made from shoulder to shoulder, and another was carried from the centre of the chin to the sternum, dividing the integuments into two flaps, which were thrown up and backward to the angles of the jaw. The muscles separated from the clavicles and sternum, the clavicles separated from their articulations; and the sternum was removed after sawing through the ribs. By these means a perfect view of the parts in their relative situations was obtained.

No vestige of the aneurismal sac remained; the artery and vein were obliterated, the former from its bifurcation, as low as its origin from the aorta, between which and the arteria innominata, a distinct aneurism of the aorta of the size of an orange was discovered; the rupture in the vessel was completely closed by organised coagulated lymph, which had formed an obstacle to the further escape of blood from it into the sac, and in consequence of which the contents of the sac had become putrid and offensive. The right carotid ar-

* The former part of this case will be found in No 23, page 165 *et seq.* of this Journal.—*Rev.*

tery had become much enlarged. Thickening of the cellular substance surrounding the vessels; extensive adhesion of lungs; watery effusion into the cavities of the chest; pericardium distended, and containing ten ounces of sero-purulent fluid; heart soft, its surface covered by a thick layer of a curdy looking purulent matter, and coagulated lymph; inner membrane of the trachea rough and thickened; the bronchiæ filled with a frothy purulent-looking fluid. An abscess containing about an ounce of ill-conditioned pus at the right angle of the lower jaw, with a destruction of the cellular substance surrounding it and the parotid gland.

The morbid appearances were such, that it is wonderful how the patient survived so long; and although the fatal termination of the case was contrary to my most sanguine expectations, the dissection satisfactorily proved the success of my operation—the artery obliterated, the sac wanting, and no communication between it and the sac of the aortic aneurism.

I have the honour to remain,
Sir,
Your most obedient humble servant,
ALEX. MONTGOMERY,
Surgeon, R.N.
And Surgeon of the Civil Hospital.

*To*
*Sir Jas. M'Gregor, Kt. &c.*
*London.*

*Remarks.* The first part of this interesting and in many points inexplicable case, will be found in the last quarterly number of this Journal, at page 165 et seq. On introducing the case to the notice of our readers and the profession, we observed that we were indebted for it to Sir James M'Gregor, to whom it was officially communicated by Mr. Montgomery. Had the particulars been published in any other medical Journal of *character*, we should have deemed it a duty to notice them, and express those critical opinions on the question they involve, which the public have in some measure a right to expect from us, and which it would be a proof of incapacity on our part to withhold. But although our own pages have been the medium of the communication, and the author has thus become in some sort our guest, we are confident that neither he nor any one will take offence, if we venture to dissent from his conclusions. If the Arab eat salt with the Giaour the latter is safe; surely if we do the same by Mr. Montgomery, though the salt be somewhat of the Attic kind, we shall 'scape as well as the unbeliever with the child of the desert. Let us try.

We cannot then sympathise with our author in his exultation or satisfaction respecting the issue of the case; we cannot agree that, "the dissection satisfactorily proved the success of the operation." On what does this proof rest? On the fact that the aneurismal sac, one too of very considerable size if it existed at all, had *entirely disappeared* when the body was examined after death. The operation ultrà tumorem is performed for an aneurism, the base of which occupies two-thirds of the sternal portion of the clavicle, whilst the superior border reaches nearly four inches upwards to the angle of the jaw, and in four short months not a vestige of the disease remains; it is gone, dissolved away,—

And like the airy fabric of a vision,
Left not a wreck behind!

We ask all who are conversant with pathology and morbid anatomy, whether such a fact squares with their experience; it certainly does not with our's. We ask all who are engaged in practice, and particularly those who have paid attention to the diseases of the heart and the arteries, whether such a fact agrees with *their* experience; again we say it is contrary to our's. It is possible, (for what is not possible in nature?) but it is not probable—it is not absolutely incredible, but yet we cannot bring ourselves to believe in it—faith is not a voluntary matter with any man, and in the present instance we have none. Credat Judæus. Having thus expressed our sentiments, it is only fair to Mr. Montgomery and to those who may differ from us in opinion, to acknowledge that the case is one of great obscurity, and that though we think there was *not* an aneurism, we cannot pretend to explain what there *was*.

But even granting that an aneurism actually existed and was removed, still we must dissent from the conclusion that the operation was successful. The object of a surgical operation, if we reason rightly, is not the mere removal of a morbid part or process *per se*, but the removal of it in such a manner that the individual may no longer suffer from the consequences of it; in other words, the *cure*, more or less perfect, of the patient. Thus if a child labour under fungus hæmatodes of the eye, and the organ be extirpated, it is clear that the disease would be removed, and the operation, according to Mr. Montgomery's reasoning, successful. If, however, we trace the progress of the case, we find that the little patient almost invariably dies, from fungus hæmatodes of the brain, of the other eye, of the cicatrix, or of the liver; in short, from a renewal of the disease in the site of the operation or elsewhere. The operation then, when we look to the *whole* of the case, is not only not successful, but is now very generally abandoned by surgeons as a desperate and cruel measure. To take another illustration;—a patient has his leg amputated for scrofulous disease of the ankle-joint, recovers from the immediate consequences of the operation, but dies in the course of two or three months of scrofulous abscesses in the lungs. The operation in this case was successful, quoad the mere disease in the joint, but unsuccessful in the extended and liberal sense of the term, for the man died of its indirect effects. It is the narrow mode of reasoning we are combating which has been the curse of physic, practitioners theorizing on separate phenomena and neglecting the general bearing of facts, till the practice of physic and surgery has grown a thing of as many shreds and patches as a beggar's coat. The surgeon will pick out his part of a case and the physician his, and between the two the case itself, the common origin of either's speculations, will be plucked as bald as the elderly man with the young and old wife.

But to return to Mr. Montgomery's case. We conceive that, even allowing an aneurism to have been removed by the operation, still the issue of the latter was neither successful nor calculated to encourage surgeons in its repetition. The patient died in four months after its performance, never having perfectly recovered in the interval; and on dissection, there were found the evidences of chronic pericarditis, an aneurismal sac on the arch of the aorta, "much enlargement" of the opposite carotid, hydrothorax, and a putrid abscess at the right angle of the lower jaw. If such be considered by surgeons an encouraging case, we are rather disposed to wonder at, than admire, their taste. It would be interesting, and to a certain extent profitable, to enquire, which of the foregoing morbid appearances were subsequent, and which antecedent in their origin to the operation. The abscess near the jaw is evidently among the former class, and probably so is the dilatation of the opposite carotid. The aneurismal sac on the arch of the aorta would appear to have been of older date, but it would be difficult to determine precisely the age of the pericarditis and hydrothorax, although we should be disposed to rank one or both in the sequelæ of the operation.

Our space is so nearly exhausted that we must waive the consideration of one or two topics connected with the case. We regret that no mention is made of the state of the coats of the aorta, thoracic and abdominal, and indeed the account of the dissection is neither so complete nor so clear as might be wished. It is merely said of the heart that it was soft; the size of its cavities, the thickness of its parietes, and the condition of its valves, not being even alluded to. We regret these omissions, we repeat, because we know, from an experience in these matters by no means inconsiderable, that aneurismal pouches and dilatations of the great vessels at the root of the neck, are in the great majority of cases combined with disease of the heart, especially hypertrophy and dilatation of its left ventricle, and with a morbid state of the coats of the aorta. We have been collecting facts on this subject which may be laid hereafter before the profession; suffice it to say, that we believe a considerable revolution in the treatment of aneurism is impending, and that fifty years hence many a bloody deed which is now performed upon the arteries as a matter of course, will *not* be perpetrated by our descendants.

